# Clinical outcomes among hospital patients with Middle East respiratory syndrome coronavirus (MERS-CoV) infection

**DOI:** 10.1186/s12879-019-4555-5

**Published:** 2019-10-22

**Authors:** Abdulrahman Mohammed G. Habib, Mohamed Abd Elghafour Ali, Baha R. Zouaoui, Mustafa Ahmed H. Taha, Bassem Sahsah Mohammed, Nazmus Saquib

**Affiliations:** 1 College of Medicine, Sulaiman Al Rajhi Colleges, P.O. Box 777, Bukayriah, Al-Qassim Zip code 51941 Saudi Arabia; 2 Buraidah Central Hospital, Buraidah, Saudi Arabia

**Keywords:** Ribavirin, Interferon alpha, MERS-CoV, Mortality

## Abstract

**Background:**

Mortality is high among patients with Middle East Respiratory Syndrome Coronavirus (MERS-CoV) infection. We aimed to determine hospital mortality and the factors associated with it in a cohort of MERS-CoV patients.

**Methods:**

We reviewed hospital records of confirmed cases (detection of virus by polymerase chain reaction from respiratory tract samples) of MERS-CoV patients (*n* = 63) admitted to Buraidah Central Hospital in Al-Qassim, Saudi Arabia between 2014 and 2017. We abstracted data on demography, vital signs, associated conditions presented on admission, pre-existing chronic diseases, treatment, and vital status. Bi-variate comparisons and multiple logistic regressions were the choice of data analyses.

**Results:**

The mean age was 60 years (SD = 18.2); most patients were male (74.6%) and Saudi citizens (81%). All but two patients were treated with Ribavirin plus Interferon. Hospital mortality was 25.4%. Patients who were admitted with septic shock and/or organ failure were significantly more likely to die than patients who were admitted with pneumonia and/or acute respiratory distress syndrome (OR = 47.9, 95% CI = 3.9, 585.5, *p*-value 0.002). Age, sex, and presence of chronic conditions were not significantly associated with mortality.

**Conclusion:**

Hospital mortality was 25%; septic shock/organ failure at admittance was a significant predictor of mortality.

## Background

The Middle East Respiratory Syndrome Coronavirus (MERS-CoV) infection is a recent and fatal disease, detected first in Saudi Arabia, where the majority of cases have so far occurred. Subsequently, it spread through the Arabian Peninsula and into neighbouring Middle Eastern countries before it became a global concern, reaching as far as the Korean Peninsula. By February 2018, its presence was detected in 27 countries worldwide, with 2144 recorded cases, out of which 750 resulted in death [1]. MERS-CoV is a contagious disease caused by C lineage of β-coronavirus. The infection can occur either through exposure with an infected animal or human [2]. Dromedary camels are believed to have been the carrier of MERS-CoV for decades as camels of the Middle Eastern region appear to be the only zoonotic host able to transmit infection to humans [3]. There is evidence of super spreading of infection (i.e., a single patient infects a disproportionate number of contacts) in MERS-CoV, and therefore, healthcare workers who provide support to infected patients are particularly vulnerable [4]. The disease exhibits a wide range of presentations at diagnosis, e.g., from no symptoms to subtle signs of pneumonia to multi-organ failure, and has the capacity to progress rapidly to cause death [5, 6]. At present, there is no effective vaccine available to prevent this fatal infection [1].

There have been both animal and human studies on treatment efficacy for MERS-CoV infection. A common form of treatment is antiviral drugs that target specific parts of the S protein in MERS-CoV. These are known as anti-MERS-CoV neutralizing monoclonal antibodies (mAbs), anti-dipeptidyl peptidase 4 (DPP4) mAbs, peptidic fusion inhibitors, siRNA, and others [7]. MERS-CoV binds with DPP4, which is found on the surface of cells in the lungs and kidneys. Protein-targeting mAbs in mice were not reported to have given in vivo protection from MERS-CoV; nevertheless, mAbs variants, including mersmab1, 2E6 and 4C2, were found to prevent entry into DPP4 cells and effectively neutralise live MERS-CoV infection in mice [8, 9]. DPP4 antagonists target the receptor-binding domain (RBD), competing with and inhibiting MERS-CoV infection. The DPP4 antagonists used in ferrets were found to be highly protective against MERS-CoV entry [10]. Multiple RBD-mAbs were found to elicit protective and therapeutic abilities against MERS-CoV infectivity in humanised DPP4 mice and other variants, as well as in rhesus monkeys [8, 11–13].

The drugs that have been tested in humans included Interferon (alpha and beta), antiviral nucleoside analogues (Ribavirin), serine protease inhibitors (Camostat), immunosuppressant (cyclosporine, mycophenolate mofetil), monoclonal antibodies, and broad-spectrum antivirals (Nitazoxanide) [14]. In one study, multiple regimens were tested including mycophenolate mofetil, Interferon alpha and beta with or without ribavirin combination, and hydrocortisone [15]. Similarly, the efficacy of Interferon-beta with lopinavir-ritonavir has been the focus of an on-going clinical trial [16]. The most widely tested regimen, however, has been Ribavirin in combination with Interferon. This regimen has been found effective in reducing the virus replication ‘in vitro’ [17]. It has also modulated the host response and improved the clinical outcome in animal experiments [18].

Clinical outcomes of MERS-Cov patients varied substantially among previous studies. For example, hospital mortality was as low as 4% in one study [19] but as high as 100% in another study [20]. In a majority of studies, the mortality ranged between 20 and 60% [6, 21–28]. Small sample sizes (5, 14, 20, and 31) in some of the included studies likely contributed in the variation of the morality estimate [6, 20, 21, 29]. Additionally, the mortality rate was influenced by the patients’ demography (e.g., age), physical conditions at admission (e.g., fever, shock, organ failure etc.), pre-existing diseases such diabetes or hypertension, or choice of treatment [26, 30].

We, therefore, present data of a larger clinical study (*n* = 63) of confirmed cases of MERS-CoV patients who were admitted to a referral hospital in the Al-Qassim region of Saudi Arabia between 2014 and 2017. Most of these patients received Ribavirin plus Interferon alpha as treatment. We ascertained their hospital mortality and assessed whether it varied by age, sex, or pre-existing comorbid conditions.

## Methods

We used the medical records of Buraidah Central Hospital (BCH) in the Al-Qassim region and adopted a retrospective cohort design for this study. BCH is the main centre in the region for the treatment of MERS-CoV infections; patients are referred here from neighbouring secondary hospitals (e.g., King Saud Hospital in Unaizah, Sulaiman Al Habib in Buraidah, and hospitals in ArRass, AlMuthnab, and Bukayriah) and tertiary hospitals (e.g., King Fahad Specialist hospital and Prince Sultan Cancer Centre in Buraidah).

The study inclusion criteria were: (1) adult (above 17 years), (2) laboratory-confirmed MERS-CoV infection with PCR (polymerase chain reaction) detection of the virus in samples taken from the respiratory tract of the patient, and (3) patients admitted to BCH between 2014 and 2017. The exclusion criteria were: (1) suspected cases of MERS-CoV without a confirmed diagnosis, and (2) pregnant women.

**Fig. 1:**
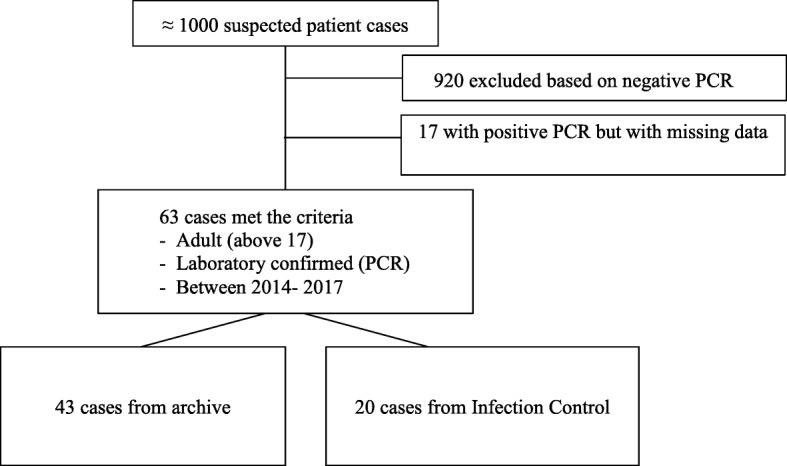
Methods

We evaluated ≈ 1000 medical records of suspected cases of MERS-CoV and excluded the ones that did not meet the eligibility criteria. The majority did not have laboratory confirmation of a MERS-CoV infection. The accuracy of data for any patient referred to BCH was crosschecked with the patient’s record from the original hospital. A total of 63 patients met the eligibility criteria and are the focus of this report. Out of 63, 43 patients’ data came from the Department of Archives at BCH; the remaining 20 came from the Department of Infection Control (from discharge summaries) at BCH since they had not yet been archived (Fig. 1). Some key information (temperature, heart rate, blood pressure, and respiratory rate) was missing from patients’ records at Infection Control. All acquired data were crosschecked with their respective patient file numbers by two co-authors to ensure there was no data duplication.

The exposure variable was the treatment regimen of Ribavirin plus Interferon alpha, which was the treatment of choice at BCH. The outcome variable was mortality during hospital stay. The covariates were age (in years), sex (male, female), nationality (Saudi, Non-Saudi), admission year (2014–2015, 2016–2017), vital signs on admission (body temperature, heart rate, respiratory rate, systolic and diastolic blood pressure), MERS-CoV associated conditions on admission (pneumonia/acute respiratory distress syndrome, septic shock/multiple organ failure, abdominal pain/diarrhoea), pre-existing comorbid conditions on admission (diabetes, hypertension, hepatitis C, chronic renal failure, and chronic heart disease), receipt of the treatment regimen (yes, no), duration of hospital stay (in days), status at discharge (alive, dead).

The Regional Ethical Committee of Al-Qassim and the administration of the BCH approved this study. The ethical committee did not require that we obtain informed consent from the patients since we examined archived medical records, had no direct contact with the patients, and ensured that the data collectors abstracted and recorded the patient data anonymously.

## Analyses

We entered and analysed the data with SPSS (version 23); we used a two-sided test with an alpha of 0.5. We calculated mean and standard deviation for continuous variables and frequency for categorical variables. From the comorbid conditions on admission, we created a summary variable with zero, one, and two or more conditions as levels. We calculated hospital mortality as the number of dead patients over the total number of patients admitted with a MERS-CoV diagnosis. We compared the hospital mortality between patients who did and did not receive the combination therapy. The observed mortality difference, the *p*-value of the test, and the sample size were used in the power calculation (power = 69%). In addition, we compared demographics, vital signs, MERS-CoV-associated conditions on admission, the number of comorbid conditions on admission, and duration of hospital stay between those who were alive and those who were dead at hospital discharge. We used Chi-square and t-test respectively for comparison of categorical and continuous variables. Finally, we used a binary multiple logistic regression model to identify correlates of hospital mortality (yes, no). We considered age, sex, admission status, and number of chronic diseases for inclusion in the model. For model robustness and ease of interpretation, we made the number of chronic diseases variable binary (no, yes). Similarly, we added the one patient who presented with abdominal pain and diarrhoea to the group with pneumonia and ARDS. We expressed the associations with odds ratio and their associated 95% confidence interval and checked model adequacy with Hosmer-Lemeshow Goodness of fit statistic.

## Results

Table 1 compares patients’ vital status by age, length of hospital stay, and vital signs on the day of admission. The sample’s mean (standard deviation) age was 59.7 (18.2) years. The majority of the patients were male (74.6%, *n* = 47) and Saudi citizens (81%, *n* = 51); the largest number of patients was admitted between 2016 and 2017 (*n* = 52) (data not shown). Hospital mortality was 25.4% (16/63); mortality among patients who received the combination therapy was 22.9% (14/61). Mean age did not differ (58.3 vs. 63.9 years; *p*-value = 0.542) between those who survived and those who died. Duration of hospital stay was significantly shorter for patients who survived (8.7 vs. 17.4 days; *p*-value < 0.0001). Mean heart rate (85.5 vs. 110.1 beats; p-value = 0.015), respiratory rate (22.1 vs. 22.8 breaths per minute; p-value = 0.004), and systolic blood pressure (127.2 vs. 128.5 mmHg; *p*-value =0.001) were significantly lower among those who survived; on the other hand, their mean diastolic blood pressure was significantly higher (70.2 vs. 63.8 mmHg; *p*-value =0.001) (comparison dead patients). Body temperature on admission did not differ between the groups (37 vs. 37.8 °C; *p*-value = 0.99).

**Table 1:** Comparison of vital status by age, duration of hospital stay, and vital signs on admission in a sample of MERS-CoV patients (*n* = 63)

Variables	N	Vital Status		*P*-value
		Alive (*n* = 47)	Dead (*n* = 16)	
		Mean (S.D.)	Mean (S.D.)	
Age^a^	63	58.3 (18.4)	63.9 (17.5)	0.542
Duration of stay in days	43	8.7 (4.3)	17.4 (21.9)	<0.001
Temperature ^°^C	43	37.0 (1.3)	37.8 (0.9)	0.992
Heart rate	43	85.5 (12.1)	110.1 (24.6)	0.015
Respiratory rate	43	22.1 (3.8)	22.8 (7.9)	0.004
Systolic blood pressure	43	127.2 (19.9)	128.5 (42.1)	0.001
Diastolic blood pressure	43	70.2 (9.6)	63.8 (22.1)	0.001

^a^available for all patients

Table 2 compares patients’ vital status in relation to their admission status, number of diseases on hospital arrival, and intake of combination regimen. Pneumonia was the most common presenting manifestation (*n* = 55, 87.3%), followed by septicaemia (*n* = 07; 11%) and abdominal pain/diarrhoea (*n* = 1; 2%). Patients who survived were more likely to have had pneumonia (95% vs. 62.5%) and less likely to have had septic shock (2.1% vs. 37.5%) at admission than patients who died (*p*-value < 0.0001). Thirty-eight percent (38%) of patients had no comorbid conditions, 14% had one condition, and 48% had two or more conditions. Patients who survived were less likely to have two or more chronic conditions than patients who died (42.6% vs. 62.5%), but the difference was not statistically significant (*p*-value = 0.13). All but two MERS-CoV patients received combination therapy of Ribavirin and Interferon (97%; 61/63). Patients who survived were more likely to have had received the combination therapy than patients who died (100% vs. 87.5%; p-value = 0.01).

**Table 2:** Comparison of vital status by admission status, number of diseases at admission, and treatment in a sample of MERS-CoV patients (*n* = 63)

Admission Status	N	Vital Status		*P*-value
		Alive N (%)	Dead N (%)	
Pneumonia / ARDS^a^	55	45 (95.7)	10 (62.5)	<0.0001
Septic Shock / MOF^b^	7	1 (2.1)	6 (37.5)	
Abdominal pain and diarrhoea	1	1 (2.1)	0 (0.0)	
N. of diseases at admission^c^				
No chronic disease	24	18 (38.3)	6 (37.5)	0.131
One chronic disease	9	9 (19.1)	0 (0.0)	
Two or more diseases	30	20 (42.6)	10 (62.5)	
Management				
Took the regimen^d^	61	47 (100.0)	14 (87.5)	0.014
Did not take the regimen	2	0 (0.0)	2 (12.5)	

^a^ARDS: Acute respiratory distress syndrome

^b^MOF: Multiple organ failure

^c^Chronic diseases included diabetes, hypertension, hepatitis C, chronic renal diseases, and chronic heart diseases

^d^All regimens included the combination therapy of Interferon alpha and Ribavirin

The odds of hospital mortality increased if the MERS-CoV patients were older or female. For example, females were 2.4 times more likely to die compared to males. On the other hand, the odds of death were lower (OR = 0.21) in patients with at least one chronic condition (reference = no chronic diseases). None of these associations, however, were statistically significant (*p* > 0.05). The only significant association was if patients were admitted to the hospitals with shock or organ failure (OR = 47.9) (reference = pneumonia/ARDS) (Table 3).

**Table 3:** Adjusted associations of hospital mortality in a sample of MERS-CoV patients (*n* = 63)

Variable	Level	N	OR	95% CI	*P*-value
Age	(in years)	63	1.03	0.99–1.07	0.19
Sex	Male	47	1.0		
	Female	16	2.4	0.49–11.6	0.28
Admission status	Pneumonia / ARDS^a^	56	1.0		
	Septic Shock / MOF^b^	07	47.9	3.9–585.5	0.002
N. of diseases at admission^c^	None	24	1.0		
	At least one	39	2.1	0.04–1.13	0.07

^a^ARDS: acute respiratory distress syndrome

^b^MOF: Multiple organ failure

^c^Chronic diseases included diabetes, hypertension, hepatitis C, chronic renal diseases, and chronic heart diseases

## Discussion

Overall, 25% of the patients died from MERS-CoV infection in our study group. Pneumonia and acute respiratory distress syndrome were the most common associated conditions that MERS-CoV patients had when they were admitted to the hospital. The patients who died had worse vital signs (such as heart and respiratory rate) at admission than patients who survived. Age, sex, or number of chronic conditions was not significantly associated with hospital mortality. Presenting with septic shock and organ failure at admission was significantly associated with hospital mortality. This was a novel finding, but the odds ratio that we reported for septic shock/organ failure should be interpreted with caution due to a very small number of patients with that condition (*n* = 07).

Three local studies reported a hospital mortality of between 22 and 28%, and an international study reported 20.4% mortality among MERS-CoV patients [19, 29–31], which is very similar to what we found in our study (25%). The similarity in mortality between these studies could be due to the aggressive and focused identification of suspected cases and dealing with them properly and early according to protocol. It should be noted that the mortality rate of our study was much lower than what other studies had reported (range 35–60%) [6, 20–28].

Our reported hospital mortality of patients who received combination therapy of Ribavirin and Interferon (22.9%) was also comparable to that of a study by Omrani et al., who reported 30% mortality among 20 patients who received the same combination therapy [29]. This mortality rate was much lower than what was reported by Al-Mekhlafi et al. (*n* = 31. 39.1%), Khaild et al. (*n* = 11, 55%), and Al-Tawfiq et al. (*n* = 5, 100%) [6, 20, 21]. The mortality with this combination therapy was found to be only 4% (1/24) in a Korean study [19]. Choi et al. attributed this reduced mortality in Koreans to the aggressive antiviral therapy, mechanical ventilatory support, and extracorporeal membrane oxygenation [19].

The variability of mortality between the studies on Ribavirin plus Interferon can be partly explained by the variability of patients’ age distribution, their physical condition, and the complications of MERS-CoV that they had at the time of admission. For example, our patients were relatively younger than patients of those studies that reported higher mortality [26, 27], and older than studies that reported a lower mortality [19]. Multiple studies found mortality increased substantially with age [26–28, 30].

Fever was the most common symptom reported in other studies and affected the majority of patients (between 69 and 98% of patients) [19, 22, 28, 30]. However, a smaller number of patients in our study were admitted with fever (defined as temperature > 37.4° = 45%). This could indicate that our patients came to the hospital at an early stage of infection, or that they used over-the-counter antipyretics before they were brought to the hospital. Finally, the studies that reported a higher mortality with the combination therapy had patients who were seriously ill with the infection; many of them were in the intensive care unit and received respiratory resuscitation by intubation or mechanical ventilation [6, 20]. Unfortunately, we lacked this critical information about our patients, and therefore, were not able to make a comparison in this respect.

Hypertension (55.6%) and diabetes (47.6%) were the most common chronic conditions among our patients; this was the case in other studies as well [26, 30]. Although the finding was not statistically significant, the direction of the association between the number of chronic conditions and mortality among MERS-CoV patients was similar to that of other studies that reported a higher mortality among those who were diabetic, hypertensive or had chronic kidney disease [26, 30].

Our study had several limitations. Although we included a relatively large number of participants, the size was still very small (*n* = 63). This could possibly explain some of the non-significant associations that we have found (e.g., sex, number of chronic conditions). We had to rely on paper-based patient records for data abstraction; some handwriting was not legible, and it is known that handwritten records are more prone to error than electronic data. In addition, the 20 patient files that were not part of the archive were very brief, and only a certain amount of information from them was usable. We did not follow patients beyond their discharge from the hospitals; hence, we could not comment on their long-term prognosis, which one study did [6]. Unlike a few other studies [21, 22], our patients almost exclusively received treatment with one regimen, i.e., Ribavirin and Interferon. The lack of a reference group (i.e., patients who received other regimens) prevented us from determining the true efficacy of this combination therapy. Finally, our study included patients 35 years or older, and therefore, we are unable to comment on the clinical outcomes in children or adolescents with MERS-CoV infection.

## Conclusion

In our sample of MERS-Cov patients, hospital mortality was 25%. Death was more likely if the patients came to the hospital with septic shock or organ failure. We were unable to compare the efficacy of the combination therapy of Ribavirin plus Interferon on mortality in the absence of patients who received other types of treatment. Future studies should strive for a larger sample, ensure inclusion of patients who received disparate therapies, and collect comprehensive information on the presenting signs and symptoms and co-exiting conditions.

## Data Availability

The datasets used and/or analysed during the current study are available from the corresponding author on reasonable request.

## Abbreviations

ARDS: Acute respiratory distress syndrome
BCH: Buraidah Central Hospital
DPP4: Anti-dipeptidyl peptidase 4
mAbs: Anti-MERS-CoV neutralising monoclonal antibodies
MERS-CoV: Middle East Respiratory Syndrome Coronavirus
MOF: Multiple organ failure
RBD: Receptor-binding domain
